# Metagenomic next-generation sequencing reveals airway microbial dysbiosis and functional alterations in *Legionella pneumophila* pneumonia

**DOI:** 10.3389/fcimb.2026.1900957

**Published:** 2026-09-22

**Authors:** Hao Zhang, Wenjing Zou, Weiwei Wu, Jun Xu, Lijun Ren, Mengying Liu, Di Sun, Jia Liu, Yanzhe Yu, Min Yu, Jingjing Ding

**Affiliations:** 1 Department of Respiratory and Critical Care Medicine, Nanjing Drum Tower Hospital, Affiliated Hospital of Medical School, Nanjing University, Nanjing, China; 2 Department of Medicine, Dinfectome Inc., Nanjing, Jiangsu, China; 3 Department of Respiratory Medicine, Lishui District People’s Hospital, Nanjing, China

**Keywords:** functional profiles, *Legionella* pneumonia, machine learning models, metagenomic next-generation sequencing, microbial characteristics

## Abstract

**Background:**

*Legionella* pneumonia (LP), primarily caused by *Legionella pneumophila*, is a clinically important subtype of community-acquired pneumonia (CAP) associated with substantial morbidity and mortality. However, the microbial characteristics and functional profiles of LP remain insufficiently understood.

**Methods:**

In this study, metagenomic next-generation sequencing (mNGS) was used to compare airway microbial communities between patients with LP and those with non-*Legionella* CAP.

**Results:**

A total of 60 patients were included, comprising 23 patients with LP patients and 37 patients with non-*Legionella* CAP patients. Airway microbial diversity and composition differed between groups, although the between-group effect size was modest and residual confounding could not be excluded. The LP group showed higher relative abundance of *Legionella pneumophila* and selected opportunistic species, whereas several oral commensal-associated species were relatively enriched in non-*Legionella* CAP. In the pooled 60-patient cohort, selected microbial species were associated with inflammatory and clinical severity markers; these associations were not disease-specific and do not imply causality. Inferred functional profiles also differed between groups. Exploratory machine-learning models showed high internal discriminatory performance, with XGBoost yielding the highest mean AUC, but the small cohort and lack of external validation limit conclusions regarding diagnostic utility.

**Conclusions:**

In this small retrospective cohort, LP was associated with differences in airway microbial composition and inferred functional potential compared with a heterogeneous non-*Legionella* CAP group. Because the study was observational, the groups differed in important clinical characteristics, and the pooled association analyses were not disease-specific, the findings should be considered exploratory and hypothesis-generating. The reproducibility and diagnostic value of microbiota-derived features require confirmation in larger, prospectively collected, independent cohorts.

## Introduction

*Legionella pneumophila* is a Gram-negative intracellular pathogen and a major cause of *Legionella* pneumonia, a clinically significant subtype of community-acquired pneumonia (CAP) (Cillóniz et al., 2016). *Legionella* pneumonia is associated with considerable morbidity and mortality, particularly among older adults, immunocompromised individuals, and patients with severe respiratory infection (Chahin and Opal, 2017; Rello et al., 2024). Because its clinical manifestations are often nonspecific and overlap with those of other CAP etiologies, early diagnosis remains challenging in routine practice (Bai et al., 2023). Delayed recognition may result in inappropriate empirical therapy and poor clinical outcomes. Therefore, improved understanding of the biological features of *Legionella* pneumonia is of substantial clinical importance.

Metagenomic next-generation sequencing (mNGS) has emerged as a powerful tool for infectious disease diagnosis and microbiological investigation (Simner et al., 2018; Gu et al., 2019; Diao et al., 2022). Unlike conventional culture-based or targeted molecular methods, mNGS enables unbiased detection of a broad spectrum of pathogens in a single assay and provides additional information on microbial community composition (Li et al., 2021). This approach is particularly valuable in pulmonary infections, where polymicrobial interactions and microbial ecological shifts may influence disease onset and progression (Montassier et al., 2023; Anani et al., 2025; Sumner et al., 2025). Accordingly, mNGS offers an opportunity not only to identify the causative pathogen but also to characterize the broader microbial landscape associated with infection.

The respiratory microbiome is increasingly recognized as an important contributor to airway homeostasis and pulmonary disease. Accumulating evidence indicates that airway microbial communities participate in host immune regulation, colonization resistance, and inflammatory signaling (Li et al., 2024; Perdijk et al., 2024). Although dysbiosis has been described in a variety of respiratory conditions, the microbial characteristics of *Legionella* pneumonia remain poorly defined, and it remains unclear whether *Legionella* pneumonia is associated with a distinct airway microbial profile beyond the presence of *Legionella pneumophila* itself. Beyond taxonomic composition, microbial functional potential may also contribute to infection-related pathogenesis, as alterations in metabolic and ecological functions may reflect adaptation to the inflamed airway environment and provide mechanistic insights into host-microbiome interactions (Hakansson et al., 2018; Di Simone et al., 2023). However, the functional features of the airway microbiota in *Legionella* pneumonia have rarely been explored. Elucidating these changes may improve our understanding of disease biology and help identify potential biomarkers for etiological discrimination or risk stratification.

In the present study, we used mNGS to characterize the airway microbiota of patients with LP and compared it with that of patients with non-*Legionella* CAP. By integrating microbiome features with clinical variables, we aimed to explore a more effective strategy for the diagnosis of *Legionella* pneumonia and to provide new insights into the clinical application of mNGS in respiratory infections.

## Method

### Participant enrollment and study design

This retrospective multicenter study enrolled patients from Nanjing Drum Tower Hospital and Lishui People Hospital between July 2021 and December 2024. Patients with clinically suspected pneumonia who underwent bronchoscopy with bronchoalveolar lavage fluid collection for routine diagnostic purposes were screened. Seventy-one patients initially met the screening criteria; 11 were excluded because complete clinical data were not available. The final cohort comprised 60 patients, including 23 with *Legionella* pneumonia and 37 with non-*Legionella* community-acquired pneumonia. Pneumonia was diagnosed based on compatible clinical symptoms, radiological evidence of pulmonary infection, and laboratory findings. Assignment to the LP group required clinical and radiological pneumonia plus microbiological evidence of *Legionella pneumophila* infection. Acceptable microbiological evidence included a positive urinary antigen test, culture, targeted PCR, serology, or mNGS. An mNGS result alone was considered sufficient supportive evidence only when *L. pneumophila* met the laboratory’s predefined positivity threshold of ≥3 reads per million non-human microbial reads (RPM) and the result was concordant with the clinical and radiological presentation. Detection below this threshold was not used for LP classification. The non-*Legionella* CAP group included patients with CAP whose available *Legionella*-specific tests were negative. Identification of an alternative pathogen was not required; therefore, this group could include other pathogen-positive, mixed, or microbiologically unresolved CAP. The inclusion criteria were: 1) age ≥18 years; 2) clinical and radiological evidence of pneumonia; 3) complete clinical data. The exclusion criteria were: 1) age <18 years; 2) severe noninfectious pulmonary diseases; 3) incomplete clinical information. Ethical approval was obtained from the Ethics Committee of Nanjing Drum Tower Hospital (2020-137-13). Written informed consent was collected from each patient upon sample collection according to the protocols approved.

### Collection of samples

Bronchoalveolar lavage fluid (BALF) samples were collected during routine diagnostic bronchoscopy at the participating centers. Briefly, the bronchoscope was advanced into the bronchial segment corresponding to the pulmonary lesion identified on chest imaging. Sterile normal saline was instilled into the target airway and then gently aspirated under negative pressure to obtain lavage fluid. The recovered BALF was transferred into sterile collection tubes and promptly sent to the laboratory for further processing. All procedures were performed under standard aseptic conditions to minimize contamination. The collected samples were sent to Nanjing Dinfectome medical laboratory for mNGS experiments, and corresponding clinical data were recorded for subsequent statistical and machine-learning analyses. Clinical and inflammatory variables were extracted from the electronic medical records. Hematological and inflammatory markers included white blood cell count (WBC), neutrophil count or percentage (Neut), lymphocyte count or percentage (Lym), platelet count (Plt), C-reactive protein (CRP), interleukin-6 (IL-6), and procalcitonin (PCT). These markers were obtained from routine clinical laboratory tests performed at admission or at the time point closest to BALF collection.

### Nucleic acid extraction

DNA was extracted using the TIANamp Magnetic DNA Kit (Tiangen) according to the manufacturer’s protocols. The quantity and quality of DNA was assessed using the Qubit (Thermo Fisher Scientific) and NanoDrop (Thermo Fisher Scientific), respectively. In parallel, no-template controls (NTCs) consisting of UltraPure DNase/RNase-free distilled water were processed using the same extraction workflow. NTC profiles were reviewed during quality control, and taxa attributable to reagent or environmental background were excluded from downstream abundance calculations and statistical analyses according to the laboratory’s validated contaminant-filtering procedures.

### Library preparation and sequencing

DNA libraries were prepared using the Hieff NGS C130P2 OnePot II DNA Library Prep Kit for MGI (Yeasen Biotechnology) according to the manufacturer’s protocols. Library quality was assessed using the Agilent 2100 Bioanalyzer. DNA libraries were sequenced as 50-bp single-end reads on the DIFSEQ-200 platform, generating approximately 20 million reads per sample. This read configuration was used because the present retrospective study relied on data generated from the routine clinical mNGS diagnostic workflow implemented at the participating laboratory.

### Bioinformatics analysis

Raw sequencing reads were processed using an in-house clinical mNGS bioinformatics pipeline. Adapter-contaminated, duplicate, low-quality, and short reads were removed using fastp version 0.23.4 with the parameters --cut_front, --cut_tail, --qualified_quality_phred 20, --length_required 36, and --dedup. Host-derived reads were identified by mapping the quality-filtered reads to the hs37d5 human reference genome using Bowtie2 version 2.5.4 with the parameters --very-sensitive-local -k 1. Reads confidently mapped to the human genome were removed, and the proportion of human-derived reads was recorded as a sequencing quality metric. The remaining non-human reads were retained for microbial taxonomic classification. The scripts used for downstream microbial community analyses are publicly available in the MicroScope repository.

A custom microbial reference database was constructed from publicly available complete and draft genome assemblies retrieved from GenBank on June 28, 2024. This database was not the complete NCBI nucleotide (nt) collection or the RefSeq database. It comprised a laboratory-curated collection of more than 12,000 bacterial, 1,000 fungal, 11,000 viral, and 300 parasitic genomes. A Kraken2 database was constructed from this curated genome collection for the initial taxonomic classification. Redundant genome records were removed on the basis of sequence similarity and taxonomic annotation to minimize duplicate representation. Taxonomic annotations were derived from the corresponding GenBank records. The database version and complete accession list were retained internally with the analysis pipeline.

Non-human reads were initially classified using Kraken2 version 2.1.3 with the parameters --confidence 0.1 and --minimum-hit-groups 2. Thus, sequence alignment was not used as the first-pass method for taxonomic screening against the entire reference collection. Instead, Kraken2 was used to identify candidate microbial taxa. For each candidate taxon identified by Kraken2, the non-human reads were subsequently aligned against the corresponding reference genomes in the curated database using Bowtie2 version 2.5.4 with the parameters --very-sensitive-local and -k 20. Up to 20 valid alignments were reported for each read to allow the identification of reads mapping to multiple closely related microbial taxa.

Alignment length was determined from the aligned portion of the read, excluding soft-clipped bases. Sequence identity was calculated from the Bowtie2 alignment output as (1 − NM/aligned query length) × 100%, where NM represents the edit distance reported in the SAM alignment record. An unambiguous species-level assignment was accepted only when the best alignment had an aligned length of at least 36 bp, a sequence identity of at least 95%, and a mapping quality (MAPQ) of at least 20. When a read had equally best-scoring valid alignments to reference genomes belonging to different taxa, it was assigned to the lowest common ancestor of those taxa rather than being assigned to an individual species. Reads that could not be resolved unambiguously at the species level were excluded from species-level quantification. A taxon was retained in an individual sample only when it was supported by at least three non-duplicate reads satisfying the applicable alignment-quality and taxonomic-assignment criteria. Moreover, for each analytical batch, taxa detected in the corresponding NTC were compiled into a batch-specific background-contaminant list (**Supplementary Table 1**) and removed from the taxonomic profiles of clinical samples processed in the same batch before abundance calculation and downstream statistical analysis.

Taxon-level abundance was normalized as reads per million non-human reads (RPM). The relative abundance of each taxon was calculated by dividing its RPM by the total microbial RPM after quality control. Prevalence was defined as the proportion of samples in which a taxon met the above taxonomic-assignment criteria. Unless otherwise specified, taxa with a mean relative abundance greater than 0.1% and a prevalence greater than 10% across all samples were retained for downstream group-level microbial community analyses.

### Microbial flora analysis

Statistical analyses were performed by R version 4.2.3. Alpha diversity was estimated using the Shannon and Simpson indices with the diversity function in vegan version 2.6-4, and group differences were assessed using the Wilcoxon rank-sum test. Beta diversity was evaluated using Bray-Curtis dissimilarity calculated with the vegdist function and visualized by principal coordinate analysis using the cmdscale. Differences in community structure between the LP and non-*Legionella* CAP groups were tested using PERMANOVA with the adonis2 function in vegan version 2.6-4. All tests were two-sided, *p* < 0.05 was considered nominally significant, and an FDR-adjusted q<0.05 was considered significant for analyses involving multiple comparisons. Exact tests and significance criteria are specified in the corresponding figure legends; descriptive composition panels were not subjected to inferential testing.

Differential taxonomic abundance was assessed using nonparametric tests in R version 4.2.3, including the Wilcoxon rank-sum test for two-group comparisons and the Kruskal-Wallis test where appropriate. Multiple testing was controlled using the Benjamini-Hochberg false discovery rate method, and an FDR-adjusted q<0.05 was considered significant. LEfSe version 1.1.2 was used to identify discriminative microbial features, with a predefined LDA score threshold of 4.5 to focus on features with relatively large discriminatory effects. For microbiota-clinical association analyses, taxa were selected from species that passed the abundance and prevalence filters and were either among the 20 most abundant species or identified as differentially represented. Spearman correlations between clinical variables and microbial relative abundances were calculated using the cor.test function in R version 4.2.3. Mantel-based network analysis was retained as an exploratory visualization of associations between selected microbial species and clinical variables, with edges defined by Mantel correlations and encoded by correlation direction, significance, and absolute R value. To assess the robustness of the LEfSe findings while accounting for the compositional nature of microbiome data, ANCOM-BC2 was performed as a complementary sensitivity analysis.

### Gene functional analysis

Functional metagenome profiling was performed using HUMAnN2 version 2.8.1 with the standard workflow and default settings. The input data were quality-filtered, host-depleted non-human reads rather than assembled metagenome-assembled genomes or a precomputed count matrix. HUMAnN2 generated UniRef90 gene family abundance tables and MetaCyc pathway abundance tables as the primary outputs. These tables were normalized to relative abundance using humann2_renorm_table, and gene-family tables were regrouped into Gene Ontology and KEGG Orthology terms using humann2_regroup_table. KEGG Orthology profiles were further mapped to KEGG pathways and modules using MinPath. Functional features were retained if they had non-zero abundance in at least 10% of samples; this filter was used to remove features confined to very few samples while preserving potentially informative functions. Differential functional features were identified using nonparametric tests with FDR correction. Functional results were interpreted as differences in inferred microbial functional potential rather than direct evidence of transcriptional or metabolic activity.

### Model construction and performance analysis

Exploratory machine-learning models were constructed to distinguish LP from non-*Legionella* CAP using selected microbial relative-abundance features and clinical variables. Clinical variables included fever, cough, chest distress or chest pain, expectoration or hemoptysis, WBC, Neut, Lym, Plt, CRP, IL-6, PCT, and time to symptom improvement. Microbial features were derived from the taxonomic abundance table after abundance and prevalence filtering, which was performed within the training set only to reduce information leakage.

Samples were randomly divided into training and testing sets at a ratio of 60:40 using the createDataPartition in caret version 6.0–94 in R version 4.2.3, and this procedure was repeated 10 times. Models were developed using the H2O framework version 3.42.0.2, including deep learning, gradient boosting machine, generalized linear model, random forest, and XGBoost. Model performance was evaluated in the held-out test sets using ROC curves generated with the roc() function in the pROC package version 1.18.5. Probability cutoffs were determined in the training set by maximizing the Youden index, calculated as sensitivity + specificity − 1 across candidate thresholds. The optimal cutoffs were 0.785 for DL, 0.357 for GBM, 0.683 for GLM, 0.644 for RF, and 0.895 for xGBoost. Predicted probabilities equal to or greater than the corresponding cutoff were classified as positive outcomes. These training-derived cutoffs were then applied unchanged to the test set to construct confusion matrices. Sensitivity, specificity, and accuracy were calculated to compare model performance in distinguishing *Legionella* pneumonia from non-*Legionella* pneumonia.

## Result

### Participant characteristics

A total of 60 patients with pneumonia were included in the final analysis, including 23 with *Legionella* pneumonia and 37 with non-*Legionella* CAP. Baseline characteristics of the study population are summarized in **Table 1**. Patients in the LP group were significantly older than those in the CAP group (*p* = 0.016). No significant differences were observed in sex, BMI, systolic blood pressure, diastolic blood pressure, heart rate, respiratory rate, or SpO2 at admission (*p*>0.05). The imbalance in age was considered a potential source of confounding in the interpretation of microbiome differences.

**Table 1:** Clinical characteristics of samples in present study.

The study cohort	Overall (n=60)	LP (n=23)	CAP (n=37)	P-value
Age, years	66 [52, 74]	73 [59.25, 77]	59 [51, 69]	0.016
Sex, male (%)	43 (71.7%)	19 (82.6%)	24 (64.9%)	0.157
BMI, kg/m²	23 ± 2.8	22.7 ± 3.1	23.1 ± 2.7	0.646
SBP, mmHg	127.9 ± 18.1	127 ± 21.7	128.3 ± 15.9	0.814
DBP, mmHg	76.3 ± 12.5	75.4 ± 16.3	76.8 ± 9.8	0.719
HR, beats/min	86.8 ± 15.7	90.7 ± 19.8	84.6 ± 12.4	0.21
RR, breaths/min	19 [18, 20]	19 [18, 22]	19 [18, 20]	0.633
SpO2, %	96.6 ± 2.3	95.9 ± 2.2	97.3 ± 2.2	0.109
Underlying disease, n (%)				
COPD	10 (16.7%)	7 (30.4%)	3 (8.1%)	0.035
Malignant tumor	10 (16.7%)	4 (17.4%)	6 (16.2%)	1
Cardiovascular and cerebrovascular diseases	20 (33.3%)	16 (69.6%)	4 (10.8%)	<0.001
Diabetes	4 (6.7%)	2 (8.7%)	2 (5.4%)	0.634
Chronic kidney disease/Rheumatic and immune diseases	8 (13.3%)	2 (8.7%)	6 (16.2%)	0.698
Clinical manifestations, n (%)				
Fever	29 (48.3%)	14 (60.9%)	15 (40.5%)	0.184
Cough	50 (83.3%)	19 (82.6%)	31 (83.8%)	1
Chest pain or chest tightness	34 (56.7%)	11 (47.8%)	23 (62.2%)	0.298
Expectoration or Hemoptysis	45 (75%)	15 (65.2%)	30 (81.1%)	0.223
CURB-65, score	1 [0, 1]	1 [1, 2]	0 [0, 1]	<0.001

LP, *Legionella* pneumonia; CAP, community-acquired pneumonia; BMI, body mass index; SBP, systolic blood pressure; DBP, diastolic blood pressure; HR, heart rate; RR, respiratory rate; COPD, Chronic obstructive pulmonary disease.

Regarding underlying diseases, chronic obstructive pulmonary disease (COPD) was more common in the LP group than in the CAP group (30.4% vs. 8.1%, *p* = 0.035). Cardiovascular and cerebrovascular diseases were also significantly more frequent in the LP group (69.6% vs. 10.8%, *p* < 0.001). No significant differences were observed in the prevalence of malignant tumor, diabetes, or chronic kidney disease/rheumatic and immune diseases (all *p*>0.05). The frequencies of major clinical manifestations, including fever, cough, chest pain or chest tightness, and expectoration or hemoptysis, were comparable between groups (all *p*>0.05). However, the CURB-65 score was significantly higher in the LP group than in the CAP group (*p* < 0.001).

### Microbial composition of BALF samples between LP and CAP patients

To characterize the microbial community structure in BALF samples from the LP and CAP groups, taxonomic profiles were analyzed at both the genus and species levels (**Figure 1**). The microbiota of all individual samples exhibited substantial inter-sample heterogeneity at the species level (**Figure 1A**). At the genus level, the LP group was mainly dominated by *Acinetobacter* (12.9%), *Legionella* (11.6%), and *Haemophilus* (8.9%), whereas *Prevotella* (16.2%), *Streptococcus* (13.4%), and *Rothia* (6.7%) were the predominant genera in the CAP group (**Figure 1B**). At the species level, the species with the highest mean relative abundances were *Legionella pneumophila* (11.7%), *Acinetobacter baumannii* (9.5%), and *Corynebacterium striatum* (6.3%) in the LP group, and *Rothia mucilaginosa* (5.6%), *Bacteroides heparinolyticus* (4.5%), and *Prevotella melaninogenica* (4.3%) in the CAP group (**Figure 1C**). These abundance summaries are descriptive; formal between-group comparisons are reported in the differential abundance analyses. The Venn diagram showed 865 shared detected species, with 588 species detected only in LP and 290 only in non-Legionella CAP (**Figure 1D**). These findings suggest that although the two groups shared a substantial microbiota, each group also possesses distinct microbial features.

**Figure 1:**
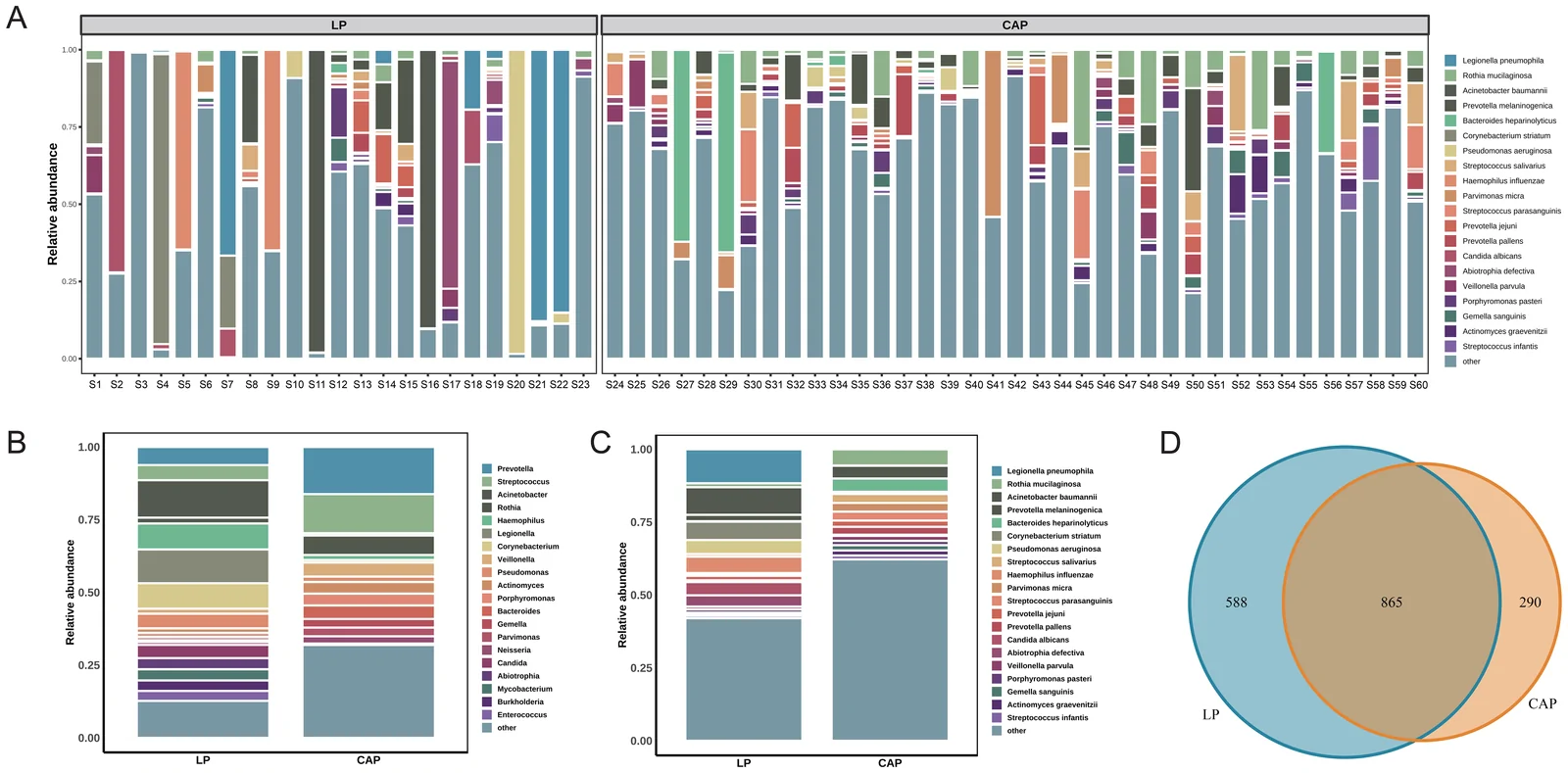
Taxonomic composition of BALF microbiota in the LP and non-*Legionella* CAP groups. **(A)** Species-level relative abundance profiles of all individual samples; **(B)** Genus-level relative abundance distribution in the two groups; **(C)** Species-level relative abundance distribution in the two groups; **(D)** Venn diagram showing shared and group-associated detected species, and full input lists are provided in **Supplementary Table 2**. Taxa were included if they passed the predefined detection threshold, with mean relative abundance >0.1% and prevalence >10% across samples. Relative abundance was calculated based on RPM-normalized non-human microbial reads after quality control and filtering. LP, *Legionella* pneumonia; CAP, community-acquired pneumonia.

### Microbial diversity analysis

Alpha diversity analysis demonstrated that microbial diversity was significantly lower in LP than in CAP (**Figures 2A, B**). Specifically, both the Shannon index (*p* = 0.00019) and Simpson index (*p* = 0.00013) were significantly decreased in the LP group compared with the CAP group. Beta-diversity analysis showed a statistically significant but modest differences in microbial community structure. Principal coordinate analysis demonstrated partial separation of the two groups, and PERMANOVA identified a significant group effect with a modest explanatory value (Adonis: R²=0.062, *p* = 0.001, **Figure 2C**). Moreover, Bray-Curtis dissimilarity analysis also revealed a highly significant difference between groups (**Figure 2D**). Collectively, these findings indicate that LP and CAP differ not only in taxonomic composition but also in within-sample diversity and overall community structure.

**Figure 2:**
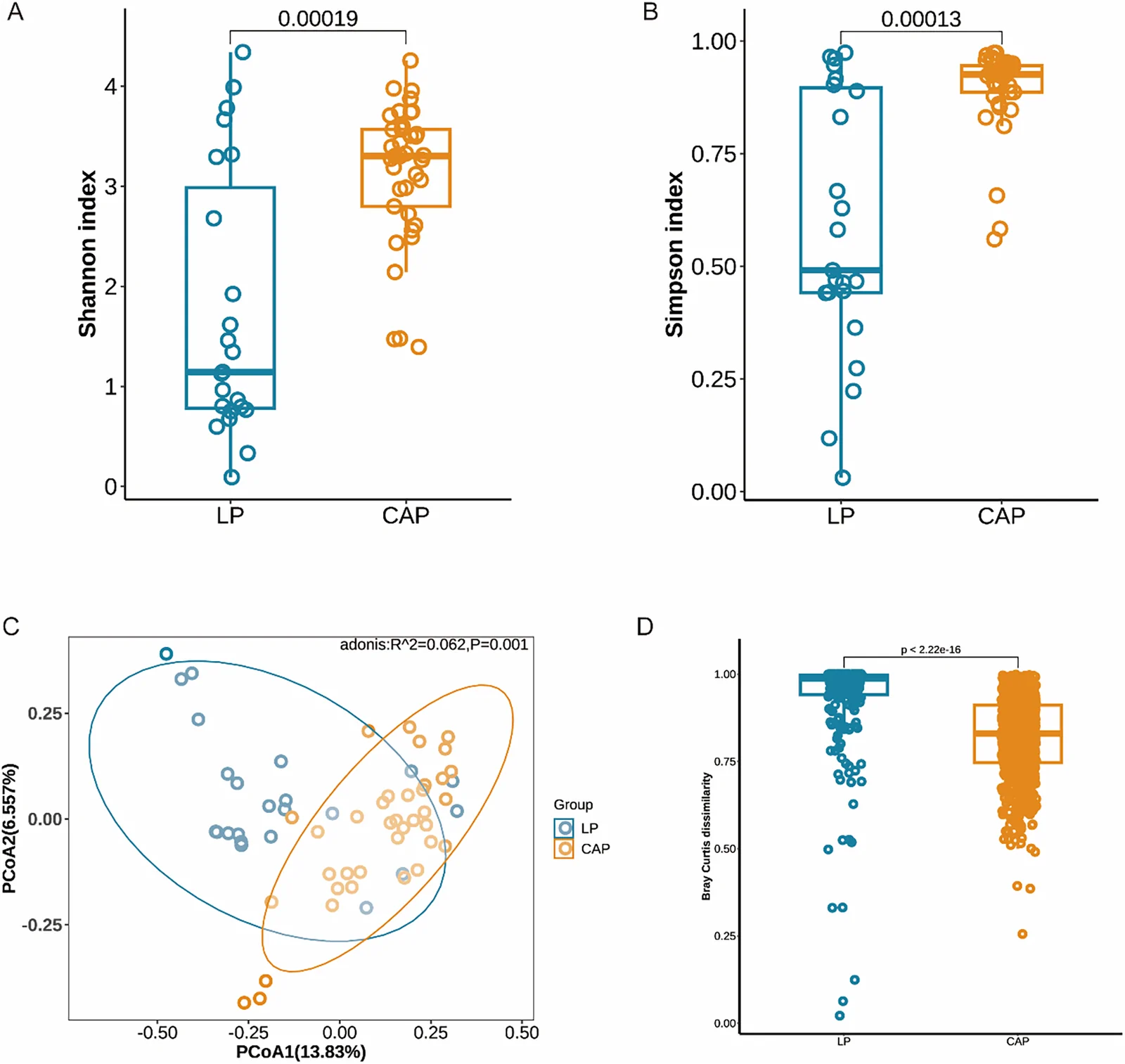
Alpha and beta diversity of BALF microbiota in the LP and non-*Legionella* CAP groups. **(A)** Shannon diversity index; **(B)** Simpson diversity index; **(C)** Principal coordinates analysis (PCoA); **(D)** Bray-Curtis dissimilarity comparison between groups. Alpha diversity differences were assessed using the Wilcoxon rank-sum test. Beta diversity differences were tested using PERMANOVA with the adonis2 function in the vegan package. The PERMANOVA result is reported as R² and p value.

### Differential species analysis between LP and CAP patients

Species-level profiling identified differences in BALF microbial composition between the LP and non-Legionella CAP groups (**Figure 3**). Heatmap analysis using identical relative-abundance thresholds showed distinct detection patterns between the two groups. *Legionella pneumophila* was detected only in the LP group and was absent from the non-*Legionella* CAP group, consistent with the predefined group classification. Several other species, including *Candida albicans* and *Acinetobacter baumannii*, were also detected in the LP group, although their detection rates gradually declined as the abundance threshold increased. In contrast, the CAP group exhibited a higher prevalence of oral commensals and anaerobic-associated species, such as *Rothia mucilaginosa*, *Prevotella melaninogenica*, *Streptococcus salivarius*, and *Streptococcus parasanguinis* (**Figures 3A, B**).

**Figure 3:**
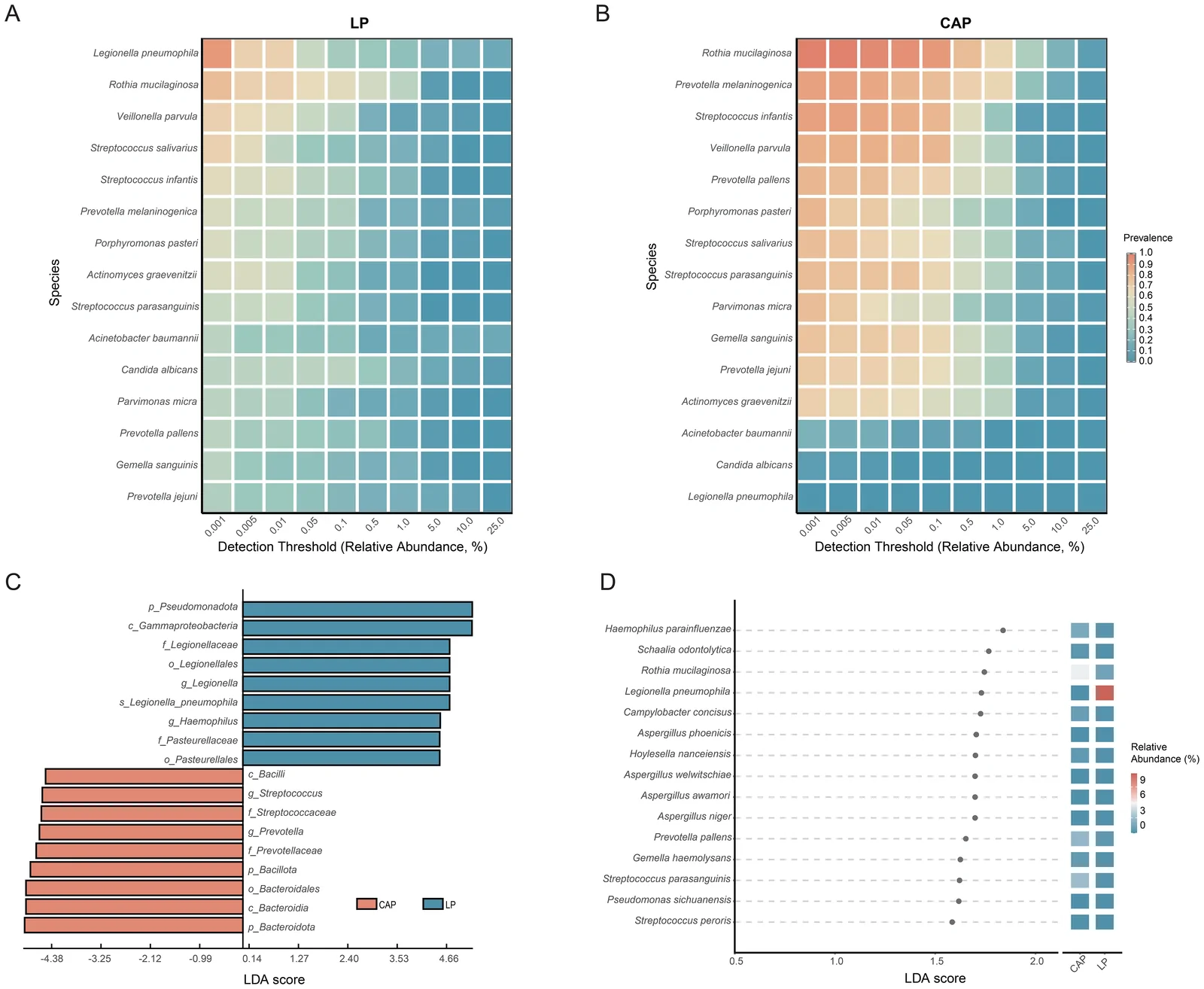
Differential microbial features between the LP and non-*Legionella* CAP groups. **(A, B)** Heatmaps showing detection rates of representative microbial species across relative-abundance thresholds. Detection rate was defined as the proportion of samples within each group in which a species exceeded the specified relative-abundance threshold; **(C, D)** LEfSe analysis identifying discriminative microbial features between groups. LEfSe was performed with an LDA score threshold of 4.5. Multiple-testing correction for differential abundance analyses was performed using the Benjamini-Hochberg false discovery rate method where applicable.

LEfSe analysis further demonstrated distinct microbial biomarkers between the two groups (**Figures 3C, D**). At the taxonomic lineage level (**Figure 3C**), the LP group was enriched in *Pseudomonadota*, *Gammaproteobacteria*, *Legionellales*, *Legionellaceae*, *Legionella*, and *Legionella pneumophila*, along with *Haemophilus*, *Pasteurellaceae*, and *Pasteurellales*. In contrast, the CAP group was enriched in *Bacteroidota*, *Bacteroidia*, *Bacteroidales*, *Bacillota*, *Prevotella*, *Prevotellaceae*, *Streptococcus*, *Streptococcaceae*, and *Bacilli*. At the species level (**Figure 3D**), *Legionella pneumophila* was predominantly enriched in the LP group, whereas the CAP group showed enrichment of several oral-associated and commensal taxa, including *Rothia mucilaginosa*, *Streptococcus parasanguinis, Prevotella pallens*, and *Haemophilus parainfluenzae*. Collectively, these results demonstrated that LP and CAP harbor distinct lower respiratory tract microbial patterns. LP was characterized by prominent enrichment of *Legionella pneumophila*, whereas CAP exhibited a more diverse microbial pattern enriched with oral-associated bacteria. As a complementary compositional analysis, ANCOM-BC2 identified additional differentially abundant species and confirmed significant enrichment of *Haemophilus influenzae* in the LP group (**Supplementary Figure 1**).

### Correlation analysis between species and clinical characteristics

The integrated correlation analysis was performed using the pooled cohort of all 60 patients (**Figure 4**). Therefore, the identified correlations represent general associations between lower respiratory tract microbiota and clinical characteristics across the combined pneumonia cohort, rather than disease-specific relationships within the LP or non-Legionella CAP group. Clinical variables included symptoms, routine hematologic indices, inflammatory biomarkers, and recovery-related measures collected from medical records. The clinical correlation matrix revealed that WBC, Neut, CRP, IL-6, and PCT were positively correlated with one another, indicating consistency among systemic inflammatory indicators. In contrast, Lym showed an inverse correlation with several inflammatory markers. Notably, PCT was positively correlated with time to symptom improvement, implying that patients with stronger inflammatory responses may experience slower clinical recovery.

**Figure 4:**
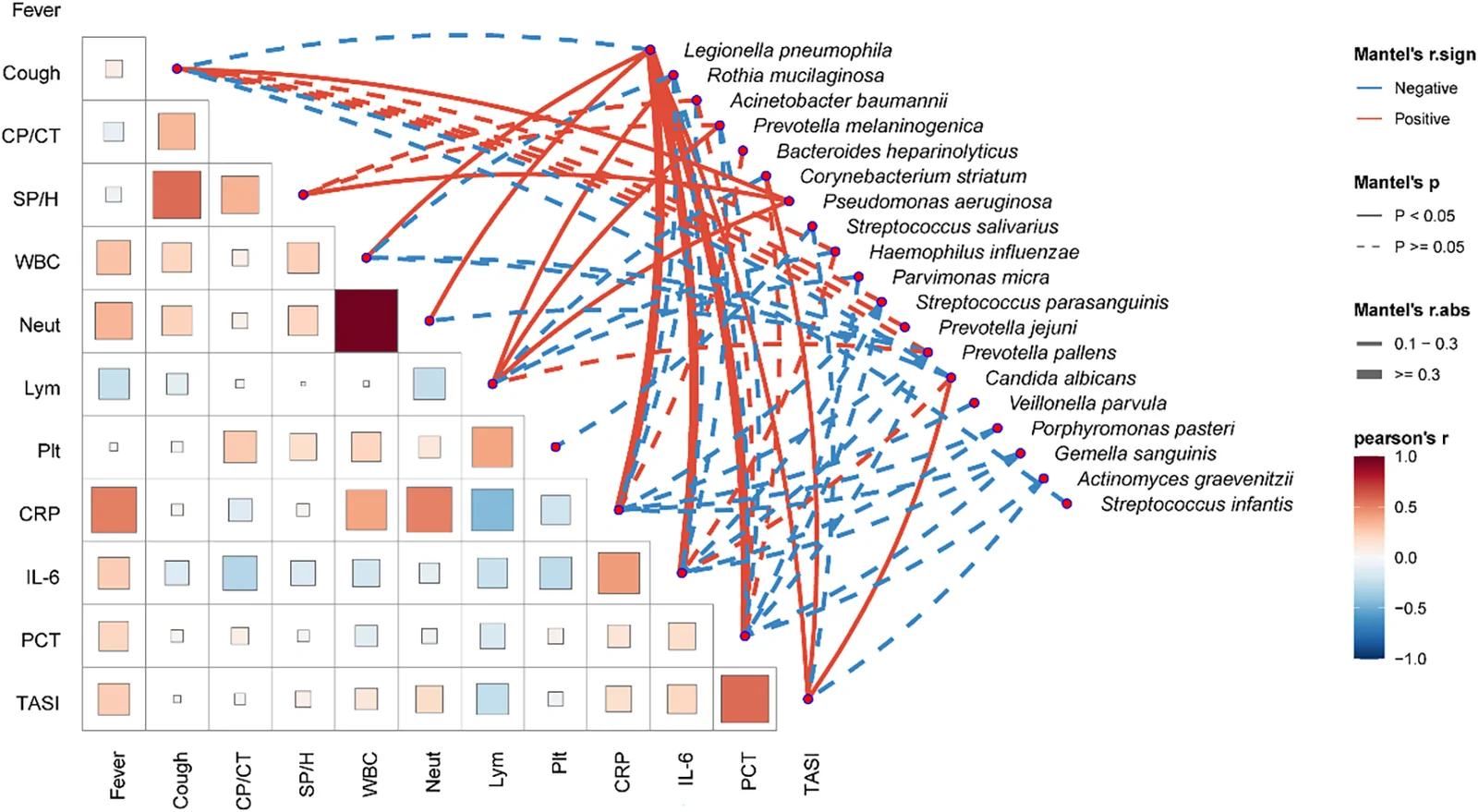
Associations between BALF microbial features and clinical characteristics. Spearman correlation analysis was used to evaluate associations between individual microbial taxa and clinical variables, and p values were adjusted using the Benjamini-Hochberg FDR method. Mantel-based network visualization was used to provide an integrated overview of the relationships between microbial community features and clinical indicators. Edge color and width indicate the direction and strength of associations, respectively. CP/CT, chest pain/chest tightness; SP/H, sputum production/hemoptysis; TASI, Time from admission to symptom improvement; WBC, white blood cell; Neut, neutrophil count; Lym, Lymphocyte count; Plt, platelet count; CRP, C-reactive protein; IL-6, interleukin-6; PCT, procalcitonin.

In the pooled analysis, several taxa relatively enriched in the LP group, including *Legionella pneumophila*, *Acinetobacter baumannii*, and *Pseudomonas aeruginosa*, tended to show positive associations with inflammatory markers such as CRP, IL-6, PCT, WBC, and Neut. In contrast, oral commensal-associated species, including *Rothia mucilaginosa*, *Prevotella melaninogenica*, *Streptococcus salivarius*, and *Veillonella parvula*, showed inverse or weaker associations with these inflammatory indicators. These pooled correlations should not be interpreted as LP- or CAP-specific microbial dynamics. Subgroup-specific correlation/Mantel analyses revealed distinct association patterns in the LP and non-Legionella CAP cohorts (**Supplementary Figure 2**). Only a subset of the associations identified in the pooled cohort remained significant after stratification, whereas several microbial–clinical associations were attenuated or no longer significant.

Species-species correlation network analysis further revealed extensive positive and negative associations among the dominant airway taxa (**Supplementary Figure 3**). *Legionella pneumophila* was associated with *Haemophilus influenzae*, *Streptococcus* spp., *Prevotella* spp., and *Veillonella parvula*, and potential pathobionts such as *Pseudomonas aeruginosa* and *Candida albicans*. These findings suggest complex microbial co-occurrence and co-exclusion patterns within the airway community. Overall, these results indicate association rather than causality between lower respiratory tract microbiota and host inflammatory phenotype.

### Differences in microbial functional gene profiles between the CAP and LP groups

Microbial functional gene analysis revealed significant differences between two groups (**Figure 5**). The LP group had a significantly higher number of annotated genes than the CAP group (*p* = 0.0019, **Figure 5A**). Venn diagram analysis showed that the two groups shared 34,719 genes, while 107,473 genes were unique to LP and 23,938 genes were unique to CAP (**Figure 5B**). After prevalence filtering, the groups showed similar major GO categories but differed in their relative functional profiles (**Figure 5C**). Individual-sample functional profiles further revealed considerable inter-patient variation within both groups (**Supplementary Figure 4**). Differential GO terms were mainly related to microbial metabolic and cellular processes, catalytic activity, and cellular structural components, with most of the displayed terms showing higher relative abundance in the LP group (**Figure 5D**). These findings indicate differences in inferred microbial functional potential and should not be interpreted as direct evidence of enhanced biological activity.

**Figure 5:**
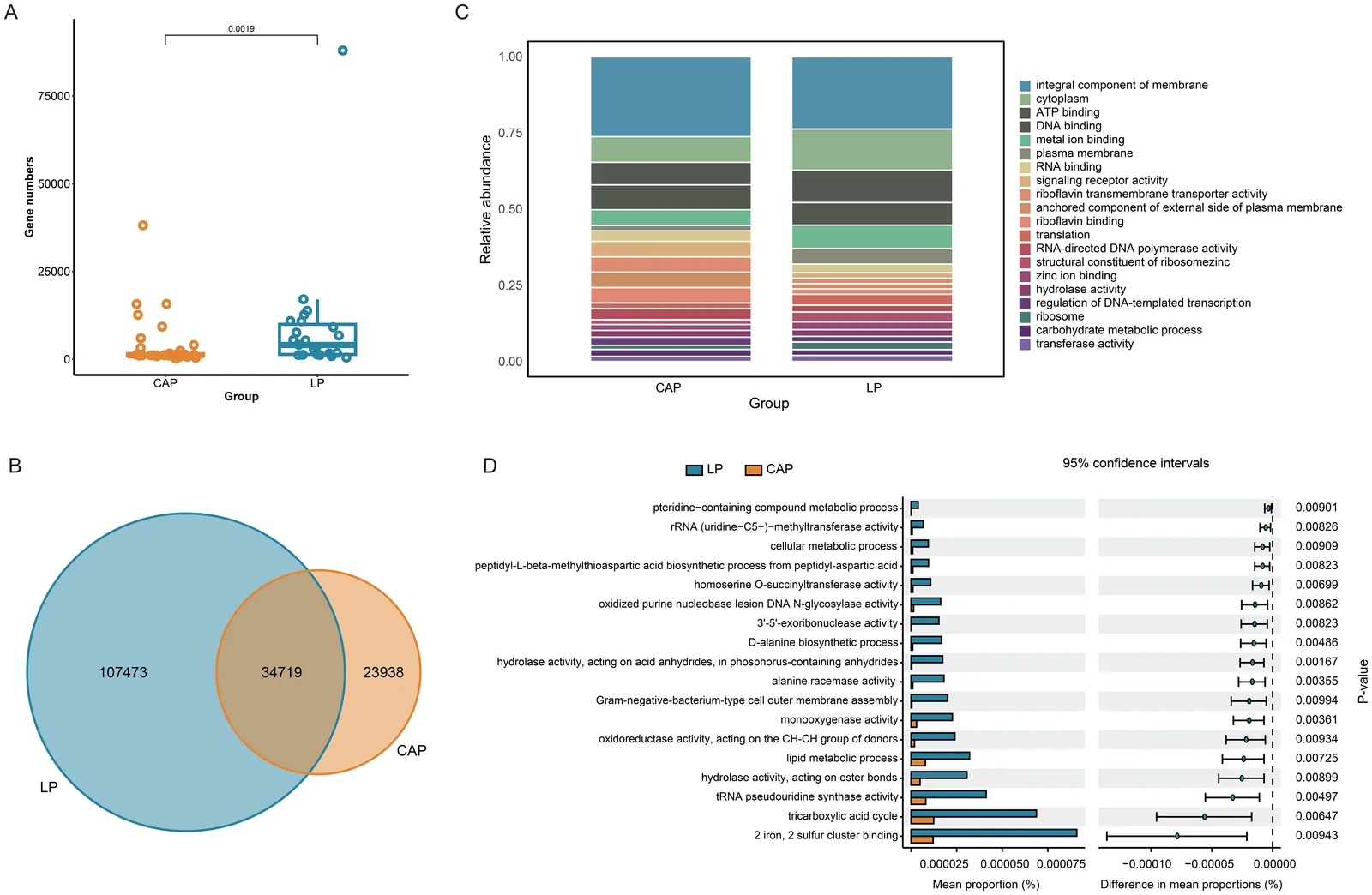
Functional gene profiling and GO annotation analysis in the CAP and LP groups. **(A)** Number of annotated microbial gene families; **(B)** Venn diagram showing shared and group-associated annotated gene families, and full input lists are provided in **Supplementary Table 3**; **(C)** Relative abundance distribution of major GO functional categories; **(D)** Differential GO terms between groups. Functional features were retained if they had non-zero abundance in at least 10% of samples. Differential comparisons were performed using nonparametric tests with Benjamini-Hochberg FDR correction.

### Comparative analysis of KEGG functional profiles between the CAP and LP groups

KEGG-based profiling was used to characterize inferred microbial functional potential from quality-filtered, host-depleted non-human reads. This analysis identified differences between the CAP and LP groups, mainly involving metabolic pathway categories, motility-related functions, secretion systems, and cell envelope-associated pathways (**Figure 6**). KEGG level 2 refers to intermediate functional pathway categories, whereas KEGG level 3 refers to more specific pathway categories within the KEGG hierarchy.

**Figure 6:**
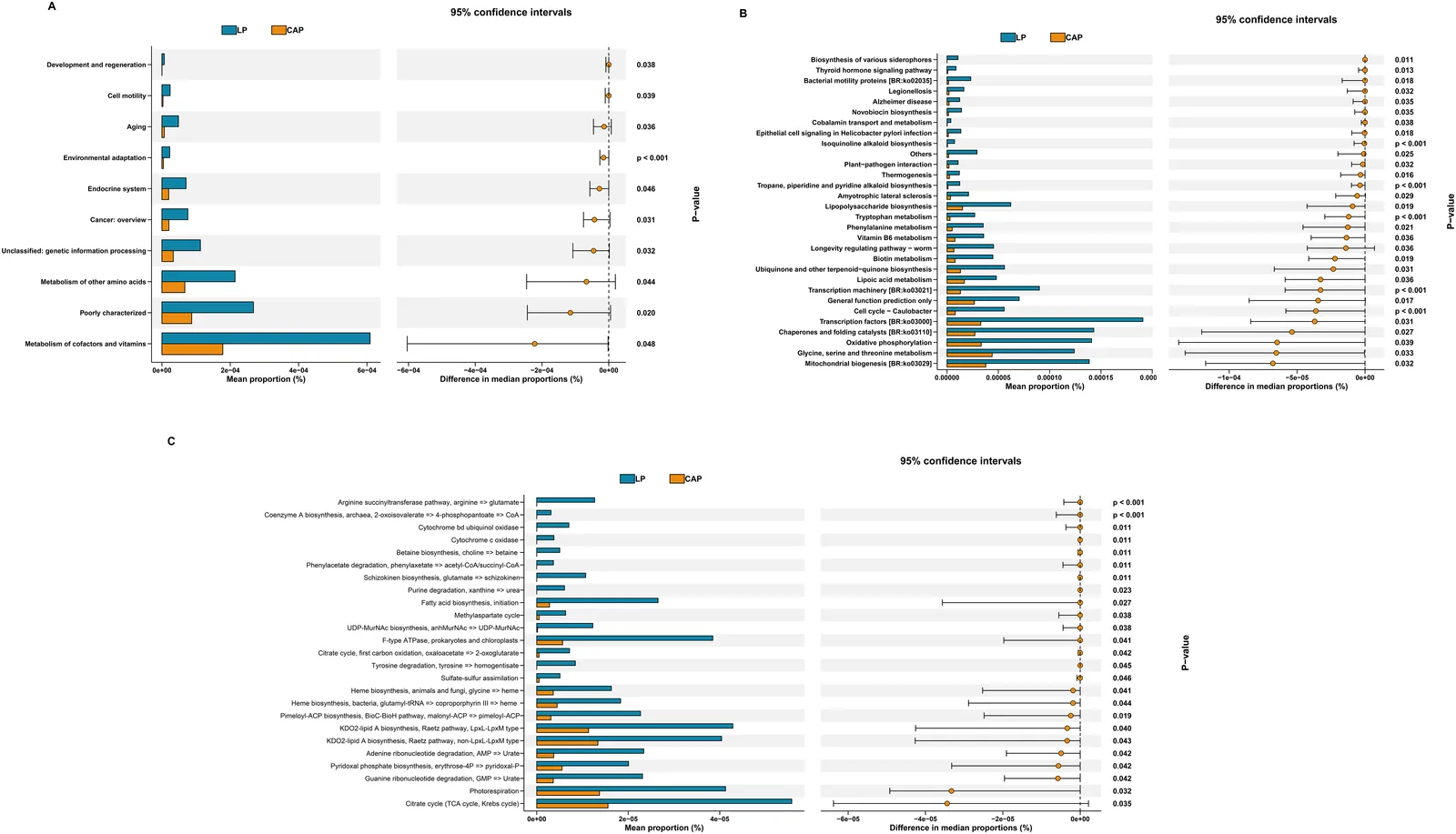
KEGG-based inferred microbial functional differences between the LP and non-*Legionella* CAP groups. **(A)** Differential KEGG level 2 pathways categories; **(B)** Differential KEGG level 3 pathways categories; **(C)** Differential KEGG modules. KEGG level 2 refers to intermediate functional pathway categories, whereas KEGG level 3 refers to more specific pathway categories within the KEGG hierarchy. Differential features were identified using nonparametric tests with Benjamini-Hochberg FDR correction.

At the KEGG level 2, the LP group exhibited significantly higher relative abundances of pathways related to metabolism of cofactors and vitamins and metabolism of other amino acids than the CAP group (**Figure 6A**). In addition, pathways associated with environmental adaptation and cell motility were also enriched in the LP group, suggesting differences in inferred microbial metabolic potential and potential responsiveness to environmental conditions. At the KEGG level 3, the LP group was significantly enriched in multiple pathways involved in amino acid metabolism, including tryptophan metabolism, phenylalanine metabolism, and glycine, serine and threonine metabolism. Notably, several pathways potentially related to immune regulation and host-microbe interactions were also significantly enriched in the LP group, such as lipopolysaccharide biosynthesis and bacterial motility proteins (**Figure 6B**).

Consistent with the pathway-level results, KEGG module analysis further demonstrated that the LP group was enriched in several modules related to amino acid metabolism and transport, including the arginine succinyltransferase pathway (M00879) and tyrosine degradation (M00044). In addition, modules involved in energy metabolism, such as the citrate cycle (M00009) and cytochrome oxidase (M00154), also differed significantly between the two groups. Notably, modules associated with lipopolysaccharide biosynthesis (M00060), lipooligosaccharide transport (M01054), and multiple secretion systems (M00958, M00959, and M00926, and M00996) were also significantly enriched in the LP group, further supporting differences in inferred microbial functional potential related to metabolism, cell envelope-associated functions, motility, and secretion systems in this group (**Figure 6C**).

### Exploratory performance of machine-learning models for distinguishing LP from non-*Legionella* CAP

To explore whether integrated microbial and clinical features could distinguish LP from non-*Legionella* CAP, we constructed exploratory models using deep learning, gradient boosting machine, generalized linear model, random forest, and XGBoost. Across 10 repeated train-test splits, XGBoost showed the highest internal discriminatory performance, with a mean AUC of 0.996 (**Figure 7**). The RF model also showed excellent performance, with an AUC of 0.9, followed by the GBM model (AUC = 0.841) and the GLM model (AUC = 0.765). In contrast, the DL model showed limited predictive performance, with an AUC of 0.544, only slightly better than random classification. The mean, standard deviation, and range of AUC, sensitivity, specificity, and accuracy across repeated splits are summarized in **Supplementary Table 4**. Overall, these exploratory results suggest that integrated microbial and clinical features may help distinguish LP from non-*Legionella* CAP in this cohort.

**Figure 7:**
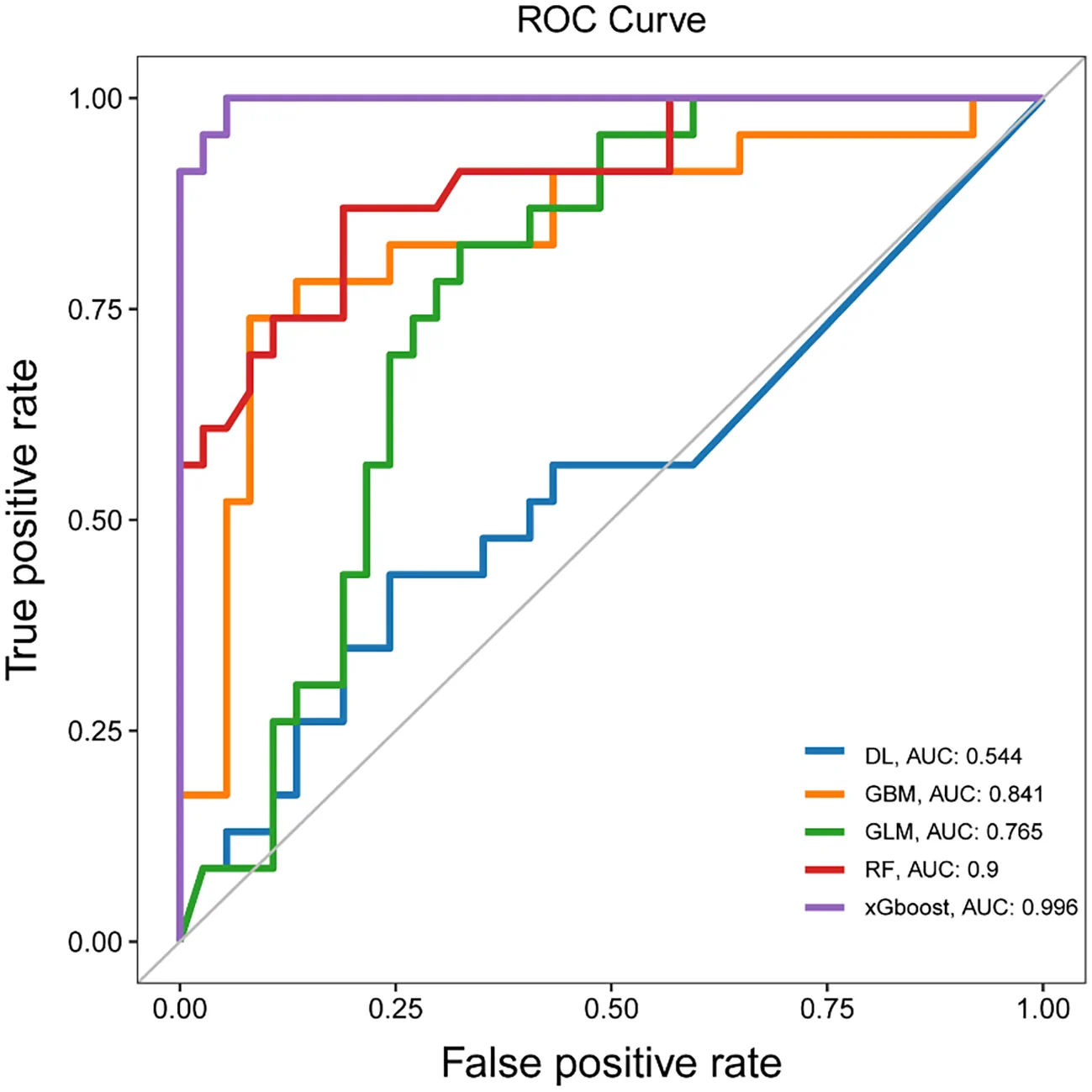
The ROC curves of machine-learning models integrating microbiome features and clinical indicators for distinguishing LP from non-*Legionella* CAP. Models were built using selected microbial relative-abundance features and clinical variables. Samples were randomly split into training and testing sets at a 60:40 ratio, repeated 10 times. ROC curves and test-set AUCs were generated using the pROC package, with probability cutoffs determined by the training-set Youden index. Given the small sample size and lack of external validation, these models are exploratory only. Performance across repeated splits is summarized in **Supplementary Table 4**.

Although XGBoost showed promising internal performance, these results should be interpreted cautiously because of the small sample size, limited test-set size, repeated holdout validation, and absence of external validation. Therefore, the models should be regarded as exploratory rather than validated diagnostic tools.

## Discussion

In this retrospective cohort, LP was associated with lower airway microbial diversity, modest but statistically significant differences in community composition, enrichment of Legionella pneumophila and selected opportunistic species, and alterations in inferred microbial functional potential compared with a heterogeneous non-*Legionella* CAP group. These findings are consistent with previous legionellosis studies reporting disturbed respiratory microbial communities, coexistence of *Legionella* with other microorganisms, and enrichment of opportunistic species during infection and antimicrobial treatment (Pérez-Cobas et al., 2020; Chotirmall et al., 2022). Differences among studies may reflect variation in specimen type, disease stage, prior treatment, sequencing strategy, and comparator composition. Accordingly, the present results should be interpreted as cohort-level LP-associated patterns rather than definitive disease-specific biology.

At the taxonomic level, the LP group showed higher relative abundance of *Legionella pneumophila* and selected opportunistic organisms, including *Acinetobacter baumannii* and *Pseudomonas aeruginosa*. This pattern may reflect multiple non-exclusive mechanisms, including disruption of epithelial and mucosal barrier function by *Legionella* infection, inflammation-driven reshaping of the airway microenvironment, and selection pressure related to prior antimicrobial exposure or therapeutic interventions (Timsit et al., 2019; Hewitt and Lloyd, 2021; Ayesha et al., 2023; Mirchandani et al., 2025). In contrast, taxa enriched outside the LP-dominant pattern were mainly oral commensal-associated organisms, such as *Rothia mucilaginosa*, *Streptococcus salivarius*, *Veillonella parvula*, and *Porphyromonas pasteri*. These microorganisms are common residents of the oral cavity and upper respiratory tract and are increasingly recognized as important components of the airway microbial ecosystem (Dong et al., 2022). Their relative depletion in LP may reflect a disruption of the normal commensal network during *Legionella* infection, whereas their relative preservation or enrichment in non-*Legionella* CAP may suggest a different ecological response pattern (Boutin et al., 2013; Pérez-Cobas et al., 2023). Collectively, our results suggest that, in this cohort, LP was associated with an altered BALF microbial profile characterized by enrichment of selected opportunistic taxa and relative depletion of commensal-associated taxa.

Besides, a distinct airway microbial signature in patients with LP compared with those with CAP was identified. Beta-diversity analysis indicated that LP is associated with a measurable alteration in airway microbiota composition. Although the explanatory effect size was modest, this is not unexpected in respiratory microbiome studies, where microbial variation is often influenced by multiple host- and treatment-related factors, including age, comorbidities, prior antibiotic exposure, inflammatory status, and disease severity (Gebrayel et al., 2022). Notably, patients with LP were older and had a higher prevalence of COPD than those with non-*Legionella* CAP. Because both age and COPD can independently affect airway microbial composition, they may have contributed to the observed differences. Given the modest sample size, robust multivariable adjustment across the high-dimensional taxonomic and functional datasets was not feasible. Therefore, these findings should be interpreted as LP-associated patterns rather than independent disease-specific effects.

The correlation analysis further indicated that airway microbial variation was closely related to host clinical manifestations and inflammatory status. Several LP-associated species, particularly *Legionella pneumophila*, *Acinetobacter baumannii*, and *Pseudomonas aeruginosa*, showed positive correlations with markers of disease activity and systemic inflammation, including fever, cough, neutrophil percentage, C-reactive protein, interleukin-6, and procalcitonin. These findings are biologically plausible, as these organisms are known to be associated with robust inflammatory responses and more severe pulmonary infection (Brown et al., 2017; Riquelme et al., 2018; Zeng et al., 2020). In contrast, species such as *R. mucilaginosa* tended to exhibit inverse associations with some inflammatory or severity-related indices, suggesting that their presence may mark a less disrupted microbial ecosystem or a comparatively attenuated inflammatory state (Hakansson et al., 2018). Although these correlations do not establish causality, they support the notion that host-microbiome interactions are an integral component of LP pathophysiology. Accordingly, the airway microbiota may serve not only as a marker of infection status but also as a potential contributor to clinical heterogeneity in disease presentation and progression (Teo et al., 2018; Li et al., 2024). The attenuation or loss of several associations after stratification suggests that some relationships observed in the pooled analysis may have been influenced by between-group differences.

Functional prediction analysis suggested that the taxonomic differences observed between LP and CAP were accompanied by alterations in inferred microbial functions. Differentially represented pathways were mainly related to metabolic processes, catalytic activity, and cellular components, indicating that the airway microbiota in LP may differ not only in composition but also in potential biological capacity (Mizrahi et al., 2017; Pérez-Cobas et al., 2020). These differences may reflect potential adaptation of microbial communities to the inflamed airway microenvironment. However, because the analysis was based on DNA sequencing, it cannot determine whether the corresponding genes were actively expressed or whether these pathways were functionally active. In addition, functional abundance tables derived from metagenomic data are inherently sparse and compositional. Although a 10% prevalence filter was applied to reduce sparsity and minimize the influence of rare functional features, zero inflation and compositional effects may still influence GO and KEGG comparisons, particularly given the limited sample size. Therefore, these findings should be interpreted cautiously as inferred functional potential rather than direct evidence of microbial activity or metabolic flux (Shima et al., 2016; Zhou et al., 2022). More direct approaches, such as deeper paired-end sequencing, metagenomics, metatranscriptomics, or metabolomics, will be necessary to validate these functional shifts and determine whether they have mechanistic relevance to LP pathogenesis (Turner and Drabovich, 2025).

The exploratory machine-learning analysis showed that microbiome-based models may capture disease-specific patterns with high accuracy and could complement conventional diagnostic approaches for *Legionella* pneumonia. This is particularly relevant because the current diagnosis of *Legionella* pneumonia often relies on urinary antigen testing, culture, or molecular methods, each of which has inherent limitations in sensitivity, specificity, or availability (Mercante and Winchell, 2015; Avni et al., 2016; Bai et al., 2023). A microbiome-based classification framework may therefore provide additional value for early recognition or etiological stratification (Marcos-Zambrano et al., 2021). Nevertheless, the cohort was small, the feature space was relatively high-dimensional, and model validation relied on repeated holdout splits without nested cross-validation or an independent external cohort. Therefore, overfitting and optimistic performance estimates cannot be excluded. The diagnostic performance and generalizability of these models should be confirmed in independent, multicenter, prospectively collected cohorts before clinical implementation can be considered.

From a broader biological perspective, the distinct microbial ecology observed in LP may reflect several non-mutually exclusive mechanisms. *Legionella pneumophila* possesses unique pathogenic features, including intracellular replication and robust activation of innate immune pathways, which may generate a host environment distinct from that seen in conventional bacterial CAP (Newton et al., 2010; Chauhan and Shames, 2021). In addition, the inflammatory and tissue-level consequences of *Legionella* pneumonia may favor microorganisms adapted to epithelial injury and immune stress (Mello-Vieira and Dikic, 2026). Prior antibiotic exposure, underlying comorbidities, disease severity, and hospital-related factors may further shape the airway microbiota (Motallebirad et al., 2024). Collectively, these factors may determine whether the airway community remains relatively commensal-rich or shifts toward pathogen dominance.

Several limitations of this study should be acknowledged. First, this was an observational, cross-sectional study, which precludes causal inference regarding the relationship between airway microbiota alterations and LP development. Second, respiratory microbiome is susceptible to confounding by prior antimicrobial exposure, corticosteroid use, smoking, comorbidities, disease severity, and specimen timing. In particular, the LP group was older and had a higher prevalence of COPD, and the limited sample size precluded robust adjustment for these imbalances across all high-dimensional analyses; residual confounding therefore cannot be excluded. Third, because the analyses were based on relative abundance data, they are inherently affected by the compositional nature of microbiome datasets and do not directly reflect absolute microbial burden. Fourth, although the machine learning models showed promising performance, their robustness and generalizability require validation in independent, multicenter, prospectively collected cohorts.

Despite these limitations, our study provides a comprehensive characterization of the airway microbiome in LP by integrating community profiling, differential taxonomic analysis, host-microbiome correlation, functional prediction, and machine learning-based classification. Collectively, these findings indicate that LP is associated with a distinct microbial ecosystem characterized by enrichment of opportunistic pathogens, depletion of commensal-associated taxa, and meaningful associations with clinical and inflammatory parameters. These findings provide exploratory insights into the airway microbiota of LP and suggest that microbiota-derived features may have potential value for etiological discrimination and disease stratification, pending validation in larger independent cohorts.

## Data Availability

The data presented in the study were deposited into the Genome Sequence Archive in the National Genomics Data Center, accession number CRA046484.
