# Supplementary material for: Metagenomic next-generation sequencing reveals airway microbial dysbiosis and functional alterations in Legionella pneumophila pneumonia
Source: Front Cell Infect Microbiol. 2026 Sep 22;16:1900957. doi: 10.3389/fcimb.2026.1900957 (PMC13638665; doi:10.3389/fcimb.2026.1900957)
Supplement: Supplementary file 1 [file DataSheet1.docx]

Supplementary Figure 1. Differentially abundant species identified by ANCOM-BC2 between the LP and non-*Legionella* CAP groups. Positive log-fold-change values indicate enrichment in the LP group, whereas negative values indicate enrichment in the CAP group. Points represent estimated log-fold changes, and error bars indicate the corresponding confidence intervals.

Supplementary Figure 2. Subgroup-specific correlation/Mantel networks in the LP and non-Legionella CAP cohorts. (A) LP cohort (n = 23). (B) Non-Legionella CAP cohort (n = 37). Spearman correlation analysis was used to evaluate associations between individual microbial taxa and clinical variables, and p values were adjusted using the Benjamini-Hochberg FDR method. Mantel-based network visualization was used to provide an integrated overview of the relationships between microbial community features and clinical indicators. Edge color and width indicate the direction and strength of associations, respectively. CP/CT, Chest pain/chest tightness; SP/H, Sputum production/hemoptysis; TASI, Time from admission to symptom improvement; WBC, White blood cell; Neut, Neutrophil count; Lym, Lymphocyte count; Plt, Platelet count; CRP, C-reactive protein; IL-6, Interleukin-6; PCT, Procalcitonin.

Supplementary Figure 3. Species-species correlation network of the airway microbiota. Nodes represent microbial species, and edges indicate pairwise correlations between species. Red edges denote positive correlations, whereas blue edges denote negative correlations. The network illustrates the co-occurrence and co-exclusion patterns of *Legionella pneumophila* with important respiratory taxa and potential pathobionts.

Supplementary Figure 4. Individual-sample functional profiles of the BALF microbiota showing the relative abundances of major Gene Ontology (GO) functional categories in the LP and non-*Legionella* CAP groups.
