# Supplementary material for: Metagenomic next-generation sequencing reveals airway microbial dysbiosis and functional alterations in Legionella pneumophila pneumonia
Source: Front Cell Infect Microbiol. 2026 Sep 22;16:1900957. doi: 10.3389/fcimb.2026.1900957 (PMC13638665; doi:10.3389/fcimb.2026.1900957)
Supplement: Supplementary file 2 [file Table1.docx]

Supplementary Table 1. Microbial species detected in the no-template controls (NTCs) and removed from the corresponding batch-specific taxonomic profiles during contaminant filtering.

| name | taxonomy_id |
| --- | --- |
| Cutibacterium acnes | 1747 |
| Corynebacterium singulare | 161899 |
| Burkholderia contaminans | 488447 |
| Ralstonia pickettii | 329 |
| Microbacterium laevaniformans | 36807 |
| Sphingomonas melonis | 152682 |
| Pseudomonas putida | 303 |
| Moraxella osloensis | 34062 |
| Sandaracinobacteroides hominis | 2780086 |
| Cupriavidus pauculus | 82633 |
| Acinetobacter johnsonii | 40214 |
| Sphingobium yanoikuyae | 13690 |
| Staphylococcus epidermidis | 1282 |
| Pseudomonas juntendi | 2666183 |
| Sphingomonas paucimobilis | 13689 |
| Aspergillus sydowii | 75750 |
| Sphingobacterium multivorum | 28454 |
| Herbaspirillum huttiense | 863372 |
| Malassezia restricta | 76775 |
| Yarrowia lipolytica | 4952 |
| Corynebacterium pseudogenitalium | 38303 |
| Saccharomyces cerevisiae | 4932 |
| Agrobacterium fabacearum | 1183401 |
| Brevibacterium casei | 33889 |
| Chryseobacterium indoltheticum | 254 |
| Ralstonia insidiosa | 190721 |
| Halomonas stevensii | 502821 |
| Micrococcus luteus | 1270 |
| Staphylococcus hominis | 1290 |
| Acinetobacter lwoffii | 28090 |
| Staphylococcus saccharolyticus | 33028 |
| Bradyrhizobium erythrophlei | 1437360 |
| Corynebacterium kefirresidentii | 1979527 |
| Staphylococcus haemolyticus | 1283 |
| Microvirgula aerodenitrificans | 57480 |
| Pseudomonas peli | 592361 |
| Staphylococcus capitis | 29388 |
| Methylobacterium radiotolerans | 31998 |
| Pseudomonas veronii | 76761 |
| Leclercia adecarboxylata | 83655 |
| Nesterenkonia massiliensis | 1232429 |
| Cladosporium cladosporioides | 29917 |
| Elizabethkingia miricola | 172045 |
| Enterobacter cloacae complex | 354276 |
| Methylobacterium jeotgali | 381630 |
| Dermabacter vaginalis | 1630135 |
| Xanthomonas campestris | 339 |
| Alternaria alternata | 5599 |
| Cutibacterium granulosum | 33011 |
| Enterobacter hormaechei | 158836 |
| Brevibacterium paucivorans | 170994 |
| Comamonas testosteroni | 285 |
| Pseudomonas stutzeri | 316 |
