# Supplementary material for: Metagenomic next-generation sequencing reveals airway microbial dysbiosis and functional alterations in Legionella pneumophila pneumonia
Source: Front Cell Infect Microbiol. 2026 Sep 22;16:1900957. doi: 10.3389/fcimb.2026.1900957 (PMC13638665; doi:10.3389/fcimb.2026.1900957)
Supplement: Supplementary file 3 [file Table4.docx]

Supplementary Table 4. Performance comparison of different machine learning models based on ROC curve analysis

| Model | Optimal cutoff | Sensitivity (%) | Specificity (%) | AUC (95% CI) |
| --- | --- | --- | --- | --- |
| DL | 0.785 | 43.5 | 75.7 | 0.544 (0.391–0.697) |
| GBM | 0.357 | 73.9 | 91.9 | 0.841 (0.728–0.954) |
| GLM | 0.683 | 82.6 | 67.6 | 0.765 (0.645–0.885) |
| RF | 0.644 | 87.0 | 81.1 | 0.900 (0.820–0.981) |
| xGBoost | 0.895 | 100.0 | 94.6 | 0.996 (0.989–1.000) |
